# Swine origin influenza A (H1N1) virus and ICU capacity in the US: Are we prepared?

**DOI:** 10.1371/currents.RRN1009

**Published:** 2009-08-22

**Authors:** Marya D. Zilberberg, Christian Sandrock, Andrew Shorr

**Affiliations:** ^*^EviMed Research Group, LLC; ^†^UC Davis and ^‡^Washington Hospital Center, Washington, DC

## Abstract

We developed a model simulating the potential impact of influenza H1N1 pandemic on the volume of acute respiratory failure requiring mechanical ventilation (ARF-MV) and the accompanying mortality rate in the US. We calculate that 46 million people will contract the infection, resulting in 2.7 million hospitalizations, 331,587 episodes of ARF-MV and nearly 200,000 deaths, suggesting that the US may require the ability to provide MV at a volume approximately 33% over the current annual use.

INTRODUCTION

In March of 2009 a novel strain of swine origin influenza A (H1N1) virus (S-OIV) was detected in Mexico. It has now spread to the rest of the world.  With over 170,000 laboratory-confirmed cases worldwide, the World Health Organization (WHO) has declared a global pandemic^[1]^. In the United States, current estimates suggest an attack rate of approximately 6-8% in affected communities, which according to officials from the Centers for Disease Control and Prevention (CDC) may more than double during the traditional flu season^[2]^. Although reports of both severe illness and fatalities are uncommon, concern exists about the potential for this virus to cause severe acute respiratory failure necessitating mechanical ventilation (ARF-MV) with specialized methods^[3]^. Since H1N1 is predicted to affect a significant proportion of the population, even a low incidence of concomitant ARF-MV may overwhelm the already-stretched emergency department and intensive care unit (ICU) resources^[4]^. To estimate what the S-OIV may portend for the ARF-MV in the US, we developed a model simulating its potential impact on the volume of ARF-MV and the accompanying mortality rate.  

METHODS

Because the situation is evolving rapidly and information is scant, in addition to peer-reviewed studies we relied on other sources of credible estimates and reasonable assumptions for model inputs (Table). We employed data from the CDC to estimate the H1N1 incidence and mortality in the US population, and those from the Mexican experience for our baseline rates of hospitalization and ARF-MV^[5]^. We explored a range of potential values for model inputs given the uncertainties surrounding them, and each outcome was subjected to 10,000 Monte Carlo simulation trials.

RESULTS AND DISCUSSION

In the base case, we calculate that 46 million people will contract the infection, resulting in 2.7 million hospitalizations, 331,587 episodes of ARF-MV and nearly 200,000 deaths (Table), suggesting that the US may require the ability to provide MV at a volume between 23% and 45% over the current use^[4]^ (Table). Tornado diagrams for each outcome indicated that the estimates are equally sensitive to all corresponding inputs (data not shown). 

Our model is limited by the accuracy of the input parameters. As the flu season progresses, more precise inputs may become available. Additionally, we did not model the impact of an effective vaccine on the attack rates or the outcomes; however, because of the uncertainties surrounding the timing and the number of doses available, and its effectiveness, this too will require updating when the information becomes available. Finally, the applicability of data from Mexico is uncertain^[5]^. Differences in the delivery of and access to healthcare between the US and Mexico may alter both rates of progression to ARF-MV and mortality.

These limitations notwithstanding, the model is informative in several ways. First, our findings represent an attempt to quantify the potential need for surge capacity in our ICUs in response to this evolving pandemic. We should point out that, although we modeled the burden over the entire epidemic period, the vast majority of the cases will likely occur during the peak flu months, thus straining the ICU and MV capacity even more intensely over a shorter period of time. For this reason, our estimates emphasize the need for careful planning and prioritization schemes as they relate to the allocation of scarce ICU resources. Second, in light of the recent report of severe ARF-MV cases requiring advanced MV modes available only in specialized centers and requiring special equipment not commonly stockpiled at the state and federal levels, planners must explore the feasibility of regionalizing S-OIV-related ARF-MV care^[3]^. Third, as this infection will likely disproportionately affect areas of higher population density, regional response plans are urgently needed.  

CONCLUSION

In summary, estimates of what may be expected with S-OIV should help ICU directors, hospital administrators and other policy makers allocate appropriate resources for an effective coordinated response. In addition to education and prevention, efforts must focus on planning for the impact of this virus on ARF-MV.

  **Table. Model input parameters and outcome estimates**

**Table:** 

**Input Parameters**	Estimate*	**Source**
US population	307,024,641	[6]
Estimated attack rate	15% (6%-24%)	[2]
Hospitalization rate, relative to attack rate	6% (2%-10%)	6% = CA experience, personal communication 2% = assumption 10% = [5]
ARF rate (relative to hospitalization)	12% (6%-18%)	12% = [5] 6% and 18% = assumption
Mortality rate (relative to ARF)	58% (40%-80%)	58% = [5] 40% and 80% =assumption
		
**Outcomes** **^ò^**	**Mean estimate**	**95% CI**
Total cases	46,053,696	36,937,583-55,094,920
Number hospitalizations	2,763,222	2,034,413-3,585,032
ARF cases	331,587	227,866-454,001
Deaths	192,320	125,945-276,482

*Each input parameter was assumed to be normally distributed

**^ò^**Outcome estimates and corresponding confidence intervals are based on Monte Carlo simulations, 10,000 trials for each outcome

CA = California, ARF = acute respiratory failure, CI = confidence interval

Acknowledgements: None

Funding information: The authors received no funding for this study

Competing interests: None

## References

[ref-543099056] Available at http://en.wikipedia.org/wiki/2009_flu_pandemic; accessed July 28, 2009

[ref-3109508557] Centers for Disease Control and Prevention (CDC) Press Brief from August 24, 2009; available at http://www.cdc.gov/media/transcripts/2009/t090724.htm; accessed July 28, 2009

[ref-827676180] Centers for Disease Control and Prevention (CDC). Intensive-care patients with severe novel influenza A (H1N1) virus infection - Michigan, June 2009. MMWR Morb Mortal Wkly Rep 2009;58:749-52 19609249

[ref-1303910377] Zilberberg MD, Shorr AF. Prolonged acute mechanical ventilation and hospital bed utilization in 2020 in the United States: implications for budgets, plant and personnel planning. BMC Health Serv Res 2008;8:242 10.1186/1472-6963-8-242 PMC2607272 19032766

[ref-1564085855] Perez-Padilla R, de la Rosa-Zamboni D, Ponce de Leon S, et al. Pneumonia and Respiratory Failure from Swine-Origin Influenza A (H1N1) in Mexico. New Engl J Med 2009 Jun 29 [Epub ahead of print] 10.1056/NEJMoa0904252 19564631

[ref-603272398] Available at http://www.census.gov; accessed July 28, 2009

